# The neighbourhood environment and profiles of the metabolic syndrome

**DOI:** 10.1186/s12940-022-00894-4

**Published:** 2022-09-03

**Authors:** Anthony Barnett, Erika Martino, Luke D. Knibbs, Jonathan E. Shaw, David W. Dunstan, Dianna J. Magliano, David Donaire-Gonzalez, Ester Cerin

**Affiliations:** 1 Mary MacKillop Institute for Health Research, Australian Catholic University, 215 Spring St, Melbourne, VIC Australia; 2 Melbourne School of Population and Global Health, The University of Melbourne, Melbourne, VIC 3010 Australia; 3 School of Public Health, The University of Sydney, Sydney, NSW 2006 Australia; 4 Department of Diabetes and Population Health, Baker Heart and Diabetes Institute, Melbourne, Australia; 5 School of Public Health and Preventive Medicine, Monash University, Melbourne, Australia; 6 Baker-Deakin Department of Lifestyle and Diabetes, Deakin University, Melbourne, Australia; 7 Department of Community Medicine, UiT The Artic University of Norway, Tromsø, Norway; 8 School of Public Health, The University of Hong Kong, 7 Sassoon Rd., Sandy Bay, Hong Kong, Hong Kong, SAR China

**Keywords:** Walkability, Greenspace, Blue space, Air pollution, Metabolic health, Neighbourhood socio-economic status

## Abstract

**Background:**

There is a dearth of studies on how neighbourhood environmental attributes relate to the metabolic syndrome (MetS) and profiles of MetS components. We examined the associations of interrelated aspects of the neighbourhood environment, including air pollution, with MetS status and profiles of MetS components.

**Methods:**

We used socio-demographic and MetS-related data from 3681 urban adults who participated in the 3rd wave of the Australian Diabetes, Obesity and Lifestyle Study. Neighbourhood environmental attributes included area socio-economic status (SES), population density, street intersection density, non-commercial land use mix, percentages of commercial land, parkland and blue space. Annual average concentrations of NO_2_ and PM_2.5_ were estimated using satellite-based land-use regression models. Latent class analysis (LCA) identified homogenous groups (latent classes) of participants based on MetS components data. Participants were then classified into five metabolic profiles according to their MetS-components latent class and MetS status. Generalised additive mixed models were used to estimate relationships of environmental attributes with MetS status and metabolic profiles.

**Results:**

LCA yielded three latent classes, one including only participants without MetS (“Lower probability of MetS components” profile). The other two classes/profiles, consisting of participants with and without MetS, were “Medium-to-high probability of high fasting blood glucose, waist circumference and blood pressure” and “Higher probability of MetS components”. Area SES was the only significant predictor of MetS status: participants from high SES areas were less likely to have MetS. Area SES, percentage of commercial land and NO_2_ were associated with the odds of membership to healthier metabolic profiles without MetS, while annual average concentration of PM_2.5_ was associated with unhealthier metabolic profiles with MetS.

**Conclusions:**

This study supports the utility of operationalising MetS as a combination of latent classes of MetS components and MetS status in studies of environmental correlates. Higher socio-economic advantage, good access to commercial services and low air pollution levels appear to independently contribute to different facets of metabolic health. Future research needs to consider conducting longitudinal studies using fine-grained environmental measures that more accurately characterise the neighbourhood environment in relation to behaviours or other mechanisms related to MetS and its components.

**Supplementary Information:**

The online version contains supplementary material available at 10.1186/s12940-022-00894-4.

## Background

The metabolic syndrome (MetS) is typically defined as a cluster of a minimum of three of five conditions: large waist circumference (WC), high levels of blood pressure (BP), fasting blood glucose (FBG) and triglycerides (TG), and low levels of high-density lipoprotein cholesterol (HDL-C) [1]. MetS prevalence increases with age [2] and has been associated with increased risk of coronary heart disease, stroke, diabetes and cancer [2, 3].

MetS is a widespread global health issue with an estimated prevalence at around 25% [1], requiring large-scale, long-term interventions targeting key modifiable risk factors. As characteristics of the environments people live in (e.g., residential neighbourhoods) have the potential to influence health-related lifestyle factors in entire populations for a sustained amount of time, it is also pertinent to study the influence of environmental attributes on MetS. This issue is particularly important and timely considering the current global increasing trends in urbanisation, densification, pollution and gentrification requiring an understanding of their possible impacts on health outcomes [4], such as MetS and its components. It also aligns with the United Nations Sustainable Development Goals targets of reducing premature mortality from non-communicable diseases through prevention (target 3.4) [5].

There is substantial evidence that the neighbourhood environment influences physical activity [6–8] and diet [9, 10], both of which have been associated with MetS [11, 12]. Also, several studies have examined associations of environmental attributes with the components of MetS [13, 14]. For example, a longitudinal study reported worsening body mass index (BMI) and WC in areas with higher dwelling density and worsening WC in areas with better access to public open space [13], while a cross-sectional study found negative associations between population density and BP [14]. Fewer studies have examined environmental correlates of MetS. One found level of urbanicity was negatively associated with MetS [15] and another reported a positive association between neighbourhood-level deprivation and MetS [16]. Others have examined associations of MetS with more fine-grain neighbourhood environment attributes, such as green space [17] and perceived land-use mix [18], as well as by-products of densification, such as air pollution, and noise [19, 20].

Overall, research in the area of neighbourhood environmental correlates of MetS typically estimated the influence of only one or a few environmental characteristics [15, 17, 21–23] and did not account for the potential causal relationships among various environmental characteristics, such as the fact that densification is a plausible antecedent of mixed land use, street connectivity and air pollution (i.e., increases in population causing the establishment of new services, road infrastructure and increases in air pollution) [24, 25]. This is a problem because focusing on one or few environmental attributes is likely to produce biased results due to unadjustment for environmental confounders. Also, disregarding the potential causal relationships among environmental attributes can lead to incorrect interpretations of the effects of these attributes on MetS. For example, the inappropriate adjustment for potential environmental mediators of the effect of an environmental exposure on MetS can result in the underestimation of its total effect and, hence, overall importance (NB: here, by total effect we refer to the sum of the effects mediated and unmediated by other environmental characteristics). Unadjustment for environmental mediators leads to biased estimates of the independent, direct (unmediated by other environmental characteristics) effects of an environmental attribute on MetS [25].

Studies on environment-health often report the results of single-environmental-attribute and multiple-environmental-attribute regression models (e.g., [6, 15]). From a causal framework viewpoint, the latter may be interpreted as direct, independent effects of the examined environmental attributes on the outcome, while the former may represent unbiased total effects (if there are no environmental causes common to the environmental attribute of interest and the outcome) or biased, confounder-unadjusted effects (if environmental causes common to both environmental attribute and outcome are not included in the model). Often, studies do not distinguish between confounder-unadjusted and total effects of environmental exposures or do not seek to estimate the total effects. This is unfortunate as important environmental determinants of health may be missed. To understand the impacts of the neighbourhood environment on metabolic health, it is important to capture the neighbourhood built, natural and socio-economic environment, the by-products of such environments (i.e., pollution) and their interrelationships, and estimate the total and direct (unmediated by other environmental characteristics) effects of each of them on MetS and its components.

Another shortcoming of previous research on environmental correlates of MetS pertains to the way MetS has been operationalised. The combination of MetS components can significantly vary within people with and without MetS, making it difficult to identify potential environmental determinants of MetS. This means that the influence of environmental characteristics on MetS may differ based on the combination of its components (e.g., high BP, TG and FBG vs. large WC, low HDL-C and high BP). It is, thus, possible that neighbourhood environmental attributes may show stronger associations with combinations of MetS components than the binary indicator of MetS status. Rather than focusing solely on the presence or absence of MetS (i.e., MetS status), determining distinct metabolic profiles that integrate information on MetS status and combinations of MetS components is likely to provide more meaningful information on the associations between neighbourhood environment characteristics and MetS. To address the above knowledge gaps, we estimated the relationships of aspects of the neighbourhood built, social and natural environment, and ambient air pollution with MetS defined in two ways: (1) the standard binary indicator of MetS status (i.e., having vs. not having MetS); and (2) metabolic profiles integrating information on MetS status and combinations of MetS components derived using latent class analysis. Rather than solely representing latent classes of MetS components, the second MetS outcome included actual information on MetS status because MetS status is a parameter of interest to clinicians and public health practitioners. This enabled the identification of environmental correlates of specific metabolic profiles with MetS vs. those without MetS. Importantly, in examining the associations of environmental attributes with MetS outcomes, we considered the potential causal relationships among various environmental attributes to estimate the total as well as direct, independent effects of each environmental attribute on the MetS outcomes.

## Methods

### Study design and participants

This study used data from wave 3 of Australian Diabetes, Obesity and Lifestyle Study (AusDiab3), a national, population-based, longitudinal cohort study of Australian adults, investigating prevalence and incidence of diabetes and associated diseases [26]. AusDiab data collection procedures have been detailed elsewhere with participants aged 25 years and over recruited from 42 statistical areas representative of Australian urban communities across the states and territories [26, 27]. The AusDiab3 study was approved by the Alfred Hospital Ethics Committee (no. 39/11). Written informed consent was obtained from all participants. The first wave was conducted in 1999–2000, while wave 3 took place in 2011–2012 [28]. A sample of 4614 participated in wave 3 and had MetS biomarker data collected at a local survey testing site [28] (41% of the first wave). Geographic Information System (GIS) data, essential for the determination of participant-specific neighbourhood environmental variables in this study, was limited to participants with a recorded address (*n* = 4141), of whom 3681 (32.7% of the first wave) had complete data. This study used only participants with complete data because the subsample with complete data was sufficiently large and the probability of having missing data was not associated with the outcome variables (i.e., having vs. not having MetS, the number of MetS criteria met and latent classes of MetS) [29]. For further information, see “Material regarding participants with complete data” in Additional file 1).

### Measures

#### Outcome variables

The criteria for presence of MetS were based on the presence of abnormal findings of three or more of the following components: 1) large WC (Caucasian: [ATP III] USA/Canada/European: men≥102 cm: women≥88 cm; Asian & Aboriginal/Torres Strait Islander: men ≥90 cm; women ≥80 cm); 2) high TG level (≥ 1.70 mmol/L) with drug treatment for elevated TG as an alternative indicator; 3) low HDL-C (men < 1.00 mmol/L; women < 1.3 mmol/L) with drug treatment for low HDL-C as an alternative indicator; 4) high BP (systolic ≥130 and/or diastolic ≥85 mmHg) with antihypertensive drug treatment as an alternative indicator; 5) elevated FBG (≥5.6 mmol/L) with drug treatment of elevated glucose as an alternative indicator [30]. For each participant, each MetS component was represented by a dichotomous variable denoting absence or presence. These five indicators were used to define latent classes (LC) of MetS components (here also named “combinations of MetS components”). Participants were then classified into metabolic profiles based on their membership to a specific latent class (i.e., combination of MetS components) and their MetS status (e.g., LC 1 with no MetS; LC 1 with MetS; LC 2 with no MetS; LC 3 with MetS, etc.). The main outcome variables were 1) MetS status (having vs. not having MetS) and 2) membership to a metabolic profile.

#### Environmental exposures

Street-network buffers (with 1-km) were created around the geocoded locations of participants’ residences following standard procedures [31]. A 1-km radius corresponds to the distance that adults and older adults without mobility problems can cover in a 10–20 minute walk [31], which is commonly used to define a neighbourhood [32].

Area socio-economic status (SES), four built environment and two natural environment measures were computed for participants’ residential buffers. The Index of Relative Socio-economic Advantage and Disadvantage (IRSAD) [33] was used to determine area SES for each participant. Built environment measures encompassed population density (persons/ha), street intersection density (intersections/km^2^) percentage of commercial land use and an entropy score denoting the heterogeneity of five non-commercial land uses (residential, industrial, medical, educational and other land uses) (Land use mix (other), range: 0–1) [34]. The two natural environment measures included in this study were percentage of parkland and percentage of blue space (e.g., lakes, coastlines, rivers and reservoirs). Exposures to nitrogen dioxide (NO_2_, units: parts per billion, ppb) and fine particulate matter smaller than 2.5 μm (PM_2.5_, units: μg/m^3^), which have been associated with MetS and its component variables (e.g., [14, 22, 35]), were estimated at each residential address using satellite-based land-use regression (LUR) models [36–38]. These models used spatial predictors of annual average NO_2_ and PM_2.5_ at fixed-site monitors (e.g., roads, industrial emissions), including time-varying information from satellites, to predict concentrations at unmeasured locations (e.g., residential addresses). The NO_2_ model captured 81% of spatial variability in annual NO_2_ (RMSE: 1.4 ppb) [36, 37], while the PM_2.5_ model captured 63% of spatial variability (RMSE: 1 μg/m^3^) [38]. The LUR models were used to predict exposure at the time of the AusDiab 3 study.

#### Covariates

Several variables were included as potential confounders in the regression models. These were self-reported sex, age, educational attainment (secondary school; trade / technician’s certificate; associate / undergraduate diploma; Bachelor’s degree or higher), household income, living arrangements (living with partner and no children; living with partner and children; living alone; other living arrangements) and tobacco smoking status (current smoked; past smoker; never smoker)*.* Two variables based on responses to 5-point-scale items assessing the importance of reasons for choosing to live in the current neighbourhood [39] were included in the regression models to account for residential self-selection (people choosing to live in neighbourhoods providing opportunities for their preferred lifestyle) [40]. One of these residential self-selection measures was related to access to recreational facilities and the other to access to various types of destinations [14, 41].

### Data analytic plan

Descriptive statistics were computed for all variables included in the study.

#### Latent class analyses

Latent class analysis (LCA) was used to identify homogenous subgroups of participants displaying specific combinations of MetS components. LCA is a type of model-based clustering operationalised by dichotomous indicators (in this case, five dichotomous variables each denoting presence or absence of a MetS component) and a categorical latent variable (denoting latent classes; in this case, combinations of MetS components). LCA derives mutually exclusive classes that maximise between-group, and minimise within-group, variance based on specific criteria of model fit [42]. With five dichotomous items (MetS components), it is theoretically possible to obtain 15 different combinations of MetS components.

Using a Bayesian approach with Gibbs sampling [43], we tested LCA models with 1 to 6 classes [44] to identify the optimal number of latent classes defining combinations of MetS components. Compared to LCA based on maximum-likelihood estimation, LCA within a Bayesian setting yields more reliable parameter estimates and standard errors especially when the probability of item endorsement (e.g., probability of having high BP in members of a specific class/combination of MetS components) approaches 0 or 1 [45]. Among the available Bayesian approaches for LCA, Gibbs sampling is considered the gold standard in terms of posterior estimation of item and latent class membership probabilities and their standard deviations [43]. The optimal number of latent classes was determined using several criteria of model fit including deviance information criterion (DIC [46];), Akaike Information Criterion Monte Carlo (AIC M[47];) Bayesian information criterion Monte Carlo (BICM [47];), sample sizes per latent class and interpretability (i.e., clear differences between latent classes). In this analytical framework, models with higher DIC, AICM and BICM values are considered to better fit the data [43]. In case of discordant results between information criteria, the model with the highest BICM values [48] providing an interpretable solution and sufficiently large classes (smallest latent class ≥5% of the sample) was selected [49, 50].

Item-response probabilities indicate the probability of having specific MetS components (e.g., high BP) conditional on the latent classes. Latent class prevalences and item response probabilities for each dichotomous indicator of MetS components were presented by latent class. Participants were classified into their respective latent classes / profiles based on their largest posterior probability of latent class membership [42]. LCAs were conducted using BayesLCA version 1.9 [43] in R version 4.0.3 [51].

#### Neighbourhood environmental correlates of MetS status and metabolic profiles

A Directed Acyclic Graph (DAG) (Additional file 1 – Fig. A1) [52], based on the hypothesised causal effects among the neighbourhood variables according to previous studies and the authors’ expert opinion, was created to determine the minimal set of confounders for regression models of total and direct, independent effects of neighbourhood environment characteristics on MetS status and membership to specific metabolic profiles. By total effect of an environmental variable, we mean confounder-adjusted association unadjusted for potential environmental mediators, while by direct, independent effect we mean confounder-adjusted association adjusted for potential environmental mediators. Confounders included in the total effects models are shown in Table 1. The direct, independent effect models were adjusted for all socio-demographic characteristics, neighbourhood self-selection variables, smoking and all environmental attributes. Generalized additive models (GAMs) accounting for clustering at the statistical-area level (by including ‘statistical area’ as a random effect term in the GAMs), and with binomial or multinomial variance and logit link functions were used to estimate these effects [53]. AIC values of GAMs with linear vs. smooth terms of environmental characteristics were compared to test curvilinearity of associations, where a ≥ 5-unit lower AIC value was indicative of a better-fitting model [54]. Graphs of curvilinear associations were presented in the Results section or supplementary material. Exponentiated regression coefficients from the derived GAMs represented odds ratios, whereby, for example, a value of 1.50 indicated that a 1-unit increase in an environmental characteristic was associated with 50% higher odds of having vs. not having MetS or belonging to a metabolic profile with MetS vs. a metabolic profile without MetS. GAMs were estimated using the package “mgcv” version 1.8–34 in R [53]. No adjustment for multiple testing was applied given that our analyses were hypothesis driven and, in this case, leading epidemiologists and statisticians consider such practice an artificial barrier to knowledge [55, 56].

**Table 1:** Potential confounders included in models of total effects of neighbourhood environment attributes on MetS status and membership to a metabolic profile

Environmental attribute	Potential confounders
Population density	*Person-level confounders:* Sex, Age, Educational attainment, Living arrangements, Employment status, Neighbourhood self-selection variables, Smoking history *Environmental confounders:* None
Commercial land use (%)	*Person-level confounders:* Same as above *Environmental confounders:* Population density
Parkland (%)	*Person-level confounders:* Same as above *Environmental confounders:* Population density, Commercial land use
Blue space (%)	*Person-level confounders:* Same as above *Environmental confounders:* None
Land use mix (other)	*Person-level confounders:* Same as above *Environmental confounders:* Population density
Street intersection density	*Person-level confounders:* Same as above *Environmental confounders:* Population density
Air pollution (NO_2_ and PM_2.5_)	*Person-level confounders:* Same as above + Household income *Environmental confounders:* Population density, Street intersection density, Commercial land use, Land use mix (other), Parkland, Area SES
Area SES (IRSAD)	*Person-level confounders:* Sex, Age, Educational attainment, Living arrangements, Employment status, Household income, Neighbourhood self-selection variables *Environmental confounders:* Parkland, Blue space

*Abbreviations: MetS* the metabolic syndrome, *SES* socio-economic status, *IRSAD* Index of Relative Socioeconomic Advantage and Disadvantage, *NO*_*2*_ nitrogen dioxide, *PM*_*2.5*_ particulate matter < 2.5 μm

Land use mix (other) represents land use excluding commercial land use, parkland and blue space Minimal sufficient adjustment sets based on the Directed Acyclic Graph (DAG)

## Results

The characteristics of the analytical sample are presented in Table 2. The mean age of participants was 60.7 years with a range from 35 to 97 years, 55% were females. Participants were relatively evenly spread across individual-level SES categories (household income and educational attainment). The mean area SES (IRSAD) was 6.4 deciles and, hence, above the average for Australia. Substantial variability in environmental attributes was observed, with, for example, population density ranging from 0.01 to 146.37 persons/ha within 1 km buffers surrounding the participants’ residential addresses. The average annual concentrations of air pollutants were low, 5.5 ppb for NO_2_ and 6.3 μg/m^3^ for PM_2.5_, respectively. The most prevalent components of MetS were large WC (73%) followed by high BP (plus those with antihypertensive drug treatment) (54%) and the least prevalent was low HDL-C (13%). Thirty three percent of the study participants had MetS.

**Table 2:** Participant characteristics (*n* = 3681)

Characteristics	Statistics	Characteristics	Statistics
*Socio-demographic and other individual and household characteristics*			
**Age, years, M ± SD**	60.7 ± 11.2	**Sex, female, %**	55.23
**Educational attainment, %**		**Smoking history, %**	
Up to secondary	32.4	Current smoker	7.2
Trade, technician certificate	29.1	Previous smoker	36.9
Associate diploma & equiv.	14.7	Non-smoker	56.0
Bachelor degree, post-graduate diploma	23.8	**Household income, annual, %**	
**Living arrangements, %**		Up to $49,999	33.6
Couple without children	49.3	$50,000 - $99,999	28.0
Couple with children	27.9	$100,000 and over	30.3
Other	22.8	Does not know or refusal	8.2
**Employment status, %**			
In paid work	54.2		
Volunteering	16.2		
Neither	29.6		
**Neighbourhood self-selection – access to destinations [range: 1–5]*,*** **M ± SD**	2.9 ± 1.3	**Neighbourhood self-selection – recreational facilities [range: 1–5]*,*** **M ± SD**	3.1 ± 1.5
*Metabolic syndrome components, including those taking drug treatments*			
**Waist circumference, %**		**Fasting blood glucose, %**	
Normal	27.3	Normal	66.3
“Obese” (circumference based on gender and ethnicity)	72.8	≥5.6 mmol/L or known diabetes on drug treatment	33.7
**Triglycerides, %**		**High-density lipoprotein–C, %**	
Normal	76.7	Normal	87.3
≥1.7 mmol/L with drug treatment for elevated triglycerides as an alternative indicator	23.3	HDL-C < 1.0 (men) < 1.3 (women) mmol/L with drug treatment for low HDL-C as alternative indicator	12.7
**Blood pressure, %**		**Number of metabolic syndrome traits per person, %**	
Normal blood pressure	45.6	0	13.0
≥130/85 mmHg with antihypertensive drug treatment as an alternative indicator	54.4	1	24.9
		2	29.1
		3	21.2
		4	9.1
		5	2.8
*Neighbourhood environment characteristics (1 km street-network buffers), M ± SD*			
**Population density, persons/ha**		**Blue space, %**	
1 km buffer	17.5 ± 10.1	1 km buffer	0.3 ± 2.1
**Commercial land use, %**		**Street intersection density, intersections/km**^**2**^	
1 km buffer	2.6 ± 6.2	1 km buffer	62.5 ± 32.7
**Parkland, %**		**Land use mix (other)**	
1 km buffer	11.7 ± 12.5	1 km buffer	0.1 ± 0.1
**Area SES (IRSAD)**	6.4 ± 2.7	**Air pollution: NO**_**2**_**, ppb**	5.6 ± 2.1
		**Air pollution: PM**_**2.5**_**, μg/m**^**3**^	6.3 ± 1.7

*Abbreviations: M* mean, *SD* standard deviation, *IRSAD* Index of Relative Socioeconomic Advantage and Disadvantage, *NO*_*2*_ nitrogen dioxide, *PM*_*2.5*_ particulate matter < 2.5 μm, *ppb* parts per billion

Values of the model fit criteria for various LCA solutions are reported in Table A1 (Additional file 1 – Table A1). A 3-class solution was deemed to best fit the data based on BICM values and sizes and interpretability of classes. The 3-class solution was associated with the highest BICM value, had latent classes defined by distinct combinations of MetS components and of acceptable size (more than 5% of the sample each). While the DIC and AICM values supported a 5-class solution (Additional file 1: Table A1), this solution had two latent classes including less than 5% of the sample and with less distinct combinations of MetS components.

**Fig. 1:**
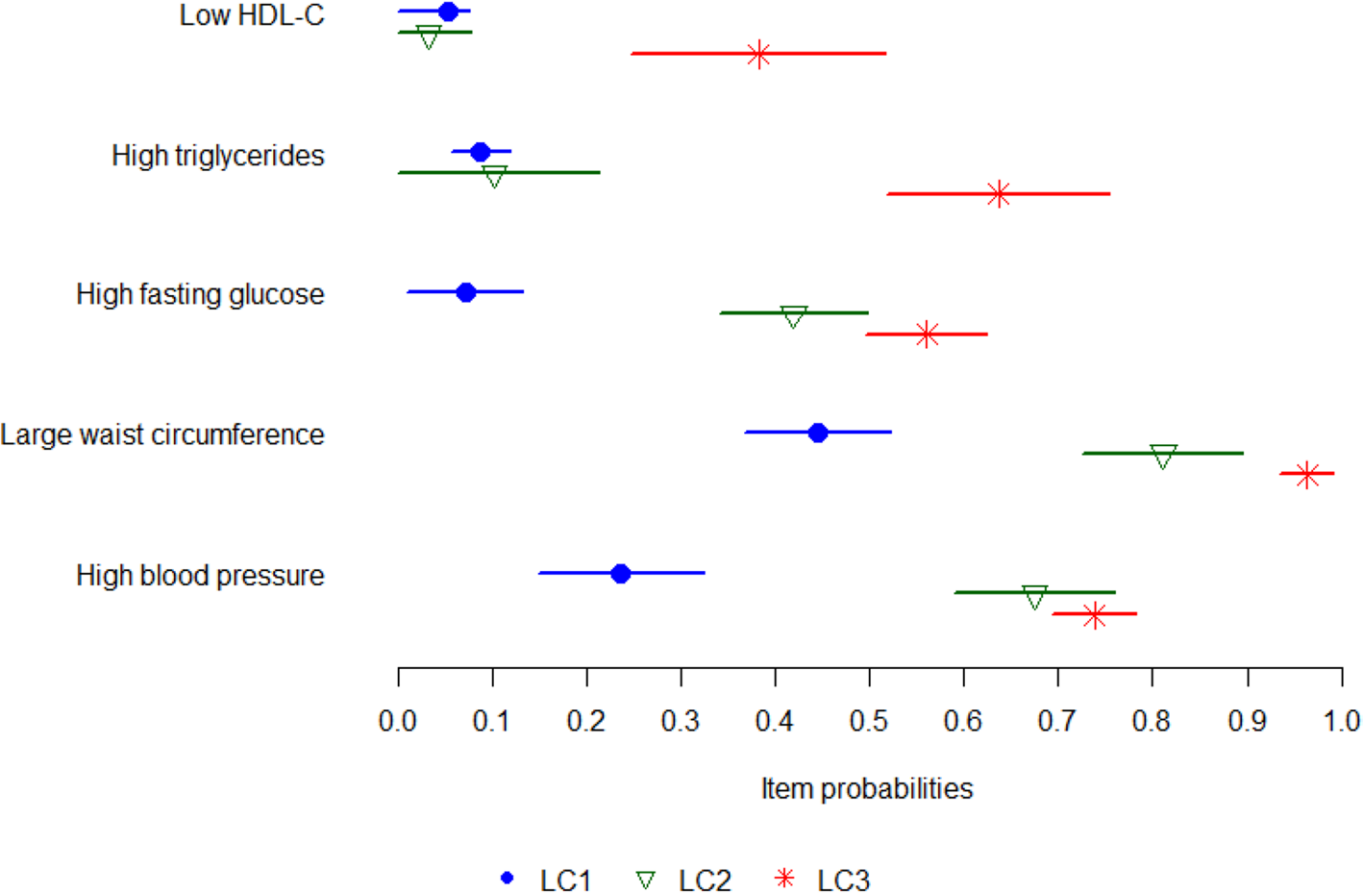
Item-response probabilities and 95% credible intervals by latent classes (LC) of metabolic syndrome (MetS) components. Legend: LC1 = Lower probability of MetS components; LC2 = Medium-to-high probability of high fasting blood glucose, waist circumference and blood pressure; LC3 = Higher probability of MetS components

Figure 1 shows the item-response probabilities by latent class (LC). LC1 (38.5% of the sample) was termed “Lower probability of MetS components” because participants falling into this class had low probability of having low HDL-C, high TG and high FBG. They also had lower probabilities than their counterparts of having a large WC and high BP. LC2 (36.3% of the sample) was named “Medium-to-high probability of high FBG, WC and BP” to highlight the differences between this LC and LC1. LC3 (25.2% of the sample) was characterised by a “Higher probability of MetS components” compared to the other LCs, as shown in Fig. 1. The three LCs differed on socio-demographic characteristics and environmental attributes (Additional file 1: Table A2), including area SES (*p* < .001) and annual average NO_2_ (*p* = .013) and PM_2.5_ (*p* = .006), with participants in the “Lower probability of MetS components” class having lower concentrations of PM_2.5_ than those in the “Higher probability of MetS component” class but higher NO_2_ concentrations than the those in the “Medium-to-high probability of high FBG, WC and BP” class. Participants with healthier LCs lived in more advantaged neighbourhoods and were more likely to be female, non-smokers and in paid work, have higher household income and education, and live with a partner (all *p*s < .001). Those in the “Lower probability of MetS components” were younger than those in the other two classes. However, those in the unhealthiest class (“Higher probability of MetS component”) were younger than those in the “Medium-to-high probability of high FBG, WC and BP” class.

Table 3 describes the metabolic profiles as combinations of the three LCs of MetS components and MetS status (having vs. not having MetS) and reports their frequencies. Among the participants not having MetS, 57.5% belonged to the “Lower probability of MetS components” LC (LC1 in Fig. 1), 38.3% to the “Medium-to-high probability of high FBG, WC and BP” LC (LC2 in Fig. 1) and 4.2% to the “Higher probability of MetS components” LC (LC3 in Fig. 1). None of the participants having MetS were classified as having “Lower probability of MetS components”. Approximately 32.2% of them fell into the “Medium-to-high probability of high FBG, WC and BP” LC (LC2 in Fig. 1) and 67.7% were classified into the “Higher probability of MetS components” LC (LC3 in Fig. 1).

**Table 3:** Metabolic profiles: description and distribution

Metabolic profile (label)	Description	n (%)
LC1 no MetS	Lower probability of MetS components & not having MetS	1417 (38.5)
LC2 no MetS	Medium-to-high probability of having high FBG, WC and BP & not having MetS	944 (25.6)
LC3 no MetS	Higher probability of MetS components & not having MetS	104 (2.8)
LC2 MetS	Medium-to-high probability of having high FBG, WC and BP & having MetS	393 (10.7)
LC3 MetS	Higher probability of MetS components & having MetS	823 (22.4)

*Abbreviations: LC* latent class, *MetS* the metabolic syndrome, *FBG* fasting blood glucose, *WC* waist circumference, *BP* blood pressure

All metabolic profiles but LC1 no MetS (defined as “Lower probability of MetS components & not having MetS”) had a high prevalence of large WC (Table 4). The patterns of prevalence of MetS components in those falling in the LC2 no MetS and LC2 MetS profiles were similar, with the only difference that those having MetS had higher prevalence of the three components characterising the profiles than those not having MetS. While prevalence of high TG and large WC was high in both LC3 no MetS and LC3 MetS profiles, those with MetS had also markedly higher prevalence of low HDL-C, high FBG and high BP (Table 4).

**Table 4:** Prevalence of MetS components within metabolic profiles

MetS components	Metabolic profiles				
	LC1 no MetS (*n* = 1417)	LC2 no MetS (*n* = 944)	LC3 no MetS (*n* = 104)	LC2 MetS (*n* = 393)	LC3 MetS (*n* = 823)
Low HDL cholesterol	6.1%	0.0%	0.0%	0.0%	**46.2%**
High triglycerides	5.4%	0.6%	**100.0%**	0.0%	**81.8%**
High fasting glucose	0.1%	**38.3%**	0.0%	**100.0%**	**58.8%**
Large waist circumference	42.3%	**83.3%**	**100.0%**	**100.0%**	**96.7%**
High blood pressure	19.0%	**70.3%**	0.0%	**100.0%**	**82.3%**

*Abbreviations: LC* latent class, *MetS* the metabolic syndrome, *HDL* high-density lipoprotein

Percentages represent the prevalence of MetS components within each of the five metabolic profiles. For example, the 19.0% prevalence of high blood pressure refers to participants falling into the LC1 no MetS profile. A description of the metabolic profiles is given in Table 3

**Fig. 2:**
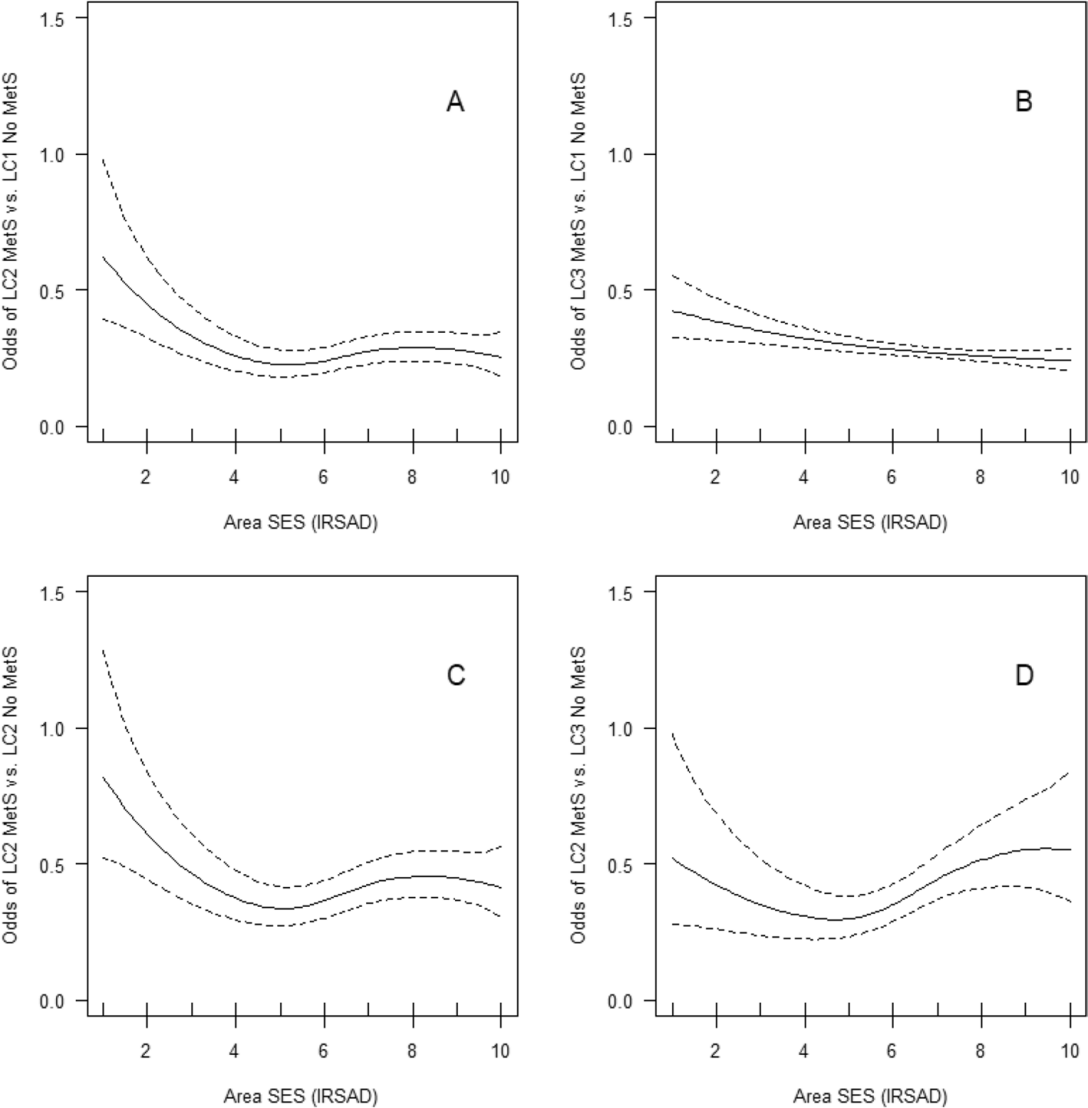
Direct effects of area SES on the odds of metabolic profiles with vs. without MetS. Legend: Panel **A**: LC2 MetS (High probability of high FBG, WC and BP & having MetS) vs. LC1 No MetS (Lower probability of MetS components & not having MetS); panel **B**: LC3 MetS (Higher probability of MetS components & having MetS) vs. LC1 No MetS; panel **C**: LC2 MetS vs. LC2 No MetS (Medium-to-high probability of high FBG, WC and BP & not having MetS); panel **D**: LC2 MetS vs. LC3 No MetS (Higher probability of MetS components & not having MetS)

Table 5 reports the total and direct effects of environmental attributes on MetS status and specific metabolic profiles with MetS vs. without MetS. Although we used GAMs with a multinomial variance function (corresponding to a multinomial regression model), here we report only the odds ratio (OR) estimates related to specific pairs of metabolic profiles of interest (i.e., metabolic profiles of participants having MetS vs. metabolic profile of participants not having MetS). Area SES was the only neighbourhood attribute significantly related to the odds of having MetS, with higher levels of area SES being associated with lower odds of MetS. For example, the total and direct effect models suggested that each 1-decile increase in area SES was approximately associated with a 4.5% (95% CI: 1.3, 7.5%; *p* = .006) and 3.9% (95% CI: 0.4, 7.3%; *p* = .027) reductions in odds of MetS, respectively. Also, there were significant linear and curvilinear total and direct effects of area SES on the odds of membership to metabolic profiles with MetS vs. without MetS (Table 5). Specifically, area SES was linearly negatively associated with the odds of membership to the least healthy metabolic profile (LC3 MetS) vs. the two healthiest metabolic profiles [LC1 No MetS (total effect model only) and LC2 No MetS]. Curvilinear associations were observed between other pairs of metabolic profiles (Fig. 2, panels A-C; Additional file 1: Fig. A2, panels A-B). An increase in area SES within the range from 1 to 5 on IRSAD was associated with lower odds of membership to the LC2 MetS profile than the two healthiest metabolic profiles (LC1 No MetS and LC2 No MetS) (Fig. 2, panels A and C; Fig. A2, panels A-B), while increases in area SES within the range from 6 to 10 on IRSAD were not related to the odds of membership to these metabolic profiles. In the fully-adjusted, direct effect model, the negative relationship between area SES and the odds of membership to the least healthy (LC3 MetS) vs the healthiest profile (LC1 no MetS) was only slightly curvilinear (Fig. 2, panel B).

**Table 5:** Neighbourhood environmental attribute associations with MetS status and metabolic health profiles with vs. without MetS (*N* = 3681)

Neighbourhood environmental attribute (T = total effect; D = direct effect)	MetS status	Metabolic health profiles					
	MetS vs. no MetS (ref.)	LC2 MetS vs LC1 no MetS (ref.)	LC3 MetS vs LC1 no MetS (ref.)	LC2 MetS vs LC2 no MetS (ref.)	LC3 MetS vs LC2 no MetS (ref.)	LC2 MetS vs LC3 no MetS (ref.)	LC3 MetS vs LC3 no MetS (ref.)
	OR (95% CI)	OR (95% CI)	OR (95% CI)	OR (95% CI)	OR (95% CI)	OR (95% CI)	OR (95% CI)
Population density (persons/ha)							
T	0.997 (0.989, 1.006)	1.001 (0.987, 1.016)	0.998 (0.988, 1.008)	1.001 (0.987, 1.016)	0.999 (0.989, 1.010)	0.997 (0.975, 1.021)	0.995 (0.974, 1.016)
D	1.001 (0.989, 1.014)	1.005 (0.984, 1.028)	0.995 (0.980, 1.009)	1.006 (0.984, 1.028)	0.995 (0.979, 1.011)	1.024 (0.988, 1.060)	1.013 (0.982, 1.045)
Commercial land use (%)							
T	0.998 (0.985, 1.011)	0.978 (0.951, 1.006)	1.010 (0.995, 1.025)	0.966 (0.940, 0.993)*	0.998 (0.984, 1.013)	0.972 (0.931, 1.014)	1.004 (0.969, 1.040)
D	0.996 (0.982, 1.009)	0.974 (0.946, 1.003)	1.007 (0.992, 1.023)	0.962 (0.935, 0.990)**	0.994 (0.979, 1.010)	0.976 (0.933, 1.021)	1.009 (0.971, 1.048)
Parkland (%)							
T	1.002 (0.996, 1.009)	1.004 (0.994, 1.014)	0.998 (0.990, 1.005)	1.009 (0.998, 1.019)	1.003 (0.995, 1.012)	0.994 (0.977, 1.011)	0.988 (0.973, 1.004)
D	1.004 (0.997, 1.011)	1.004 (0.994, 1.015)	1.000 (0.993, 1.008)	1.008 (0.997, 1.019)	1.003 (0.995, 1.012)	0.992 (0.975, 1.010)	0.988 (0.972, 1.004)
Blue space (%)							
T	0.976 (0.936, 1.018)	0.943 (0.857, 1.037)	0.982 (0.939, 1.028)	0.947 (0.861, 1.041)	0.987 (0.940, 1.036)	0.970 (0.837, 1.123)	1.011 (0.895, 1.142)
D	0.980 (0.939, 1.022)	0.960 (0.877, 1.050)	0.987 (0.942, 1.033)	0.964 (0.881, 1.055)	0.993 (0.945, 1.043)	0.985 (0.846, 1.147)	1.014 (0.889, 1.156)
Land use mix (other)							
T	1.392 (0.734, 2.641)	**2.342 (0.867, 6.338).**	1.505 (0.742, 3.052)	1.947 (0.701, 5.405)	1.383 (0.653, 2.929)	1.708 (0.295, 9.894)	1.208 (0.246, 5.943)
D	1.031 (0.514, 2.069)	1.785 (0.613, 5.194)	0.850 (0.397, 1.818)	2.137 (0.728, 6.279)	1.064 (0.476, 2.380)	3.802 (0.563, 25.670)	1.862 (0.322, 10.765)
Street intersection density (/km^2^)							
T	1.001 (0.998, 1.004)	1.002 (0.997, 1.008)	1.004 (1.000, 1.007)*	0.998 (0.993, 1.004)	1.000 (0.996, 1.003)	0.996 (0.987, 1.004)	0.997 (0.990, 1.004)
D	1.000 (0.997, 1.003)	1.001 (0.995, 1.007)	1.001 (0.998, 1.005)	0.999 (0.993, 1.005)	0.999 (0.995, 1.003)	0.995 (0.986, 1.004)	0.995 (0.987, 1.003)
Area SES (IRSAD)							
T	0.955 (0.925, 0.987)**	curvilinear** ***p*** **= .0090**	0.928 (0.894, 0.964)***	curvilinear* ***p*** **= .0496**	0.961 (0.924, 0.999)*	1.035 (0.943, 1.136)	1.024 (0.944, 1.112)
D	0.961 (0.927, 0.996)*	curvilinear** ***p*** **= .0077**	curvilinear* ***p*** **= .0101**	curvilinear* ***p*** **= .0165**	0.957 (0.917, 0.999)*	curvilinear* ***p*** **= .0239**	1.042 (0.953, 1.140)
Air pollution: NO_2_ (ppb)							
T	0.999 (0.946, 1.055)	0.991 (0.895, 1.098)	0.987 (0.928, 1.049)	1.060 (0.957, 1.173)	**1.062 (0.993, 1.136).**	0.891 (0.773, 1.027)	**0.891 (0.793, 1.001).**
D	0.995 (0.942, 1.048)	0.992 (0.895, 1.100)	0.981 (0.923, 1.042)	1.064 (0.960, 1.179)	**1.060 (0.991, 1.133).**	0.897 (0.777, 1.034)	0.889 (0.791, 0.999)*
Air pollution: PM_2.5_ (μg/m^3^)							
T	1.049 (0.993 1.108)	0.992 (0.869, 1.131)	1.070 (1.005, 1.138)*	0.955 (0.844, 1.082)	1.030 (0.963, 1.103)	0.930 (0.782, 1.107)	1.005 (0.880, 1.147)
D	1.048 (0.992, 1.107)	0.991 (0.867, 1.132)	1.072 (1.007, 1.140)*	0.950 (0.837, 1.077)	1.026 (0.959, 1.098)	0.937 (0.786, 1.117)	1.014 (0.887, 1.159)

*Abbreviations: MetS* the Metabolic Syndrome, *LC* latent class, *NO*_*2*_ nitrogen dioxide, *PM*_*2.5*_ particulate matter < 2.5 μm, *ppb* parts per billion, *SES* socio-economic status, *IRSAD* Index of Relative Socioeconomic Advantage and Disadvantage, *ref.* reference category

Land use mix (other) encompassed land use excluding commercial land use, parkland and blue space. A description of the metabolic health profiles is given in Table 3. Minimal sufficient adjustment sets of covariates for each neighbourhood attribute were based on the directed acyclic graph (DAG) (see Additional file 1: Fig. A1 and Table 1). *p* < .10; * *p* < .05; ** *p* < .01; *** *p* < .001. Significant curvilinear associations are presented in Fig. 2 and A2

When examining the odds of being in the healthiest metabolic profile (LC1 No MetS) versus the two least healthy profiles (LC2 MetS and LC3 MetS), three additional environmental correlates emerged. In the total effect models, higher land use mix levels tended to be associated with higher odds of being in the LC2 MetS profile, and higher street intersection density was predictive of higher odds of belonging to the LC3 MetS profile, than the LC1 No MetS profile. However, these associations were no longer significant after adjustment for other environmental attributes. Higher average annual concentration of PM_2.5_ was also related to higher odds of LC3 MetS than LC1 No MetS membership in both total and direct effect models.

Apart from area SES, two other environmental attributes distinguished participants falling into the LC2 No MetS profile from those in the two metabolic profiles with MetS. These were percentage of commercial land use, which was negatively associated with the odds of LC2 MetS, and average annual NO_2_, which tended to be positively related to the likelihood of LC3 MetS.

The comparisons of participants falling into the LC3 No MetS profile versus those falling under the two profiles with MetS (LC2 MetS and LC3 MetS) yielded the lowest number of environmental correlates and the weakest associations. In this instance, area SES was not positively related to the likelihood of being in the healthier profile (LC3 No MetS) (Fig. 2, panel D) and NO_2_ tended to be associated with higher odds of being in the LC3 No MetS than LC3 MetS profile. Population density and percentages of parkland and blue space were not significantly associated with MetS status or metabolic profiles with vs. without MetS.

## Discussion

Due to MetS being any cluster of at least three of five health-related conditions (large WC, high BP, high FBG, high TG and low HDL-C), it is a construct defined by two heterogenous groups of individuals: those with and without MetS. The substantial heterogeneity of these two groups in combinations of MetS components is evident from the results of the latent class analyses. In line with a recent study in a US adult sample [57], we identified three metabolic profiles of participants without MetS and two profiles with MetS. The metabolic profiles without MetS ranged from individuals with low probability of most MetS components to individuals with a relatively high probability of high BP, FBG and large WC, to those with a very high probability of large WC and high TG. Among those having MetS, we found a group without dyslipidaemia (nearly nil probability of low HDL and/or high TG) and a group with higher probability of dyslipidaemia as well as other MetS components. A similar finding has been previously observed in women [57]. Furthermore, in line with an earlier study [58], elevated blood glucose and blood pressure tended to co-occur across profiles. In this study, the only metabolic profile with a high probability of low HDL-C (LC3 MetS) exhibited relatively high probabilities (> 0.58) of all other MetS components. In this regard, recent longitudinal studies have reported decreases in HDL-C to be strong predictors of increased risk of MetS [59, 60] and be associated with the other four components [59].

Because, as evidenced in this study, MetS status categories consist of individuals with various profiles of MetS components and the impact of environmental attributes on specific MetS components may differ [61, 62], we hypothesised that fewer environmental attributes would be related to MetS status than to metabolic profiles defined using latent classes of MetS components and MetS status. We also expected environmental correlates of membership to metabolic profiles with and without MetS to differ across latent classes. The data supported both hypotheses, demonstrating the added utility of operationalising MetS as a set of profiles of MetS components using latent class analysis in studies of neighbourhood environmental determinants of metabolic health.

### Area SES

After adjustment for individual-level SES, area SES was the only significant predictor of MetS status, with participants living in more affluent areas being less likely to have MetS. In general, higher area SES was also predictive of membership to healthier (without MetS) than less healthy (with MetS) metabolic profiles. Although we are not aware of studies that examined the association of area SES with MetS or related profiles, previous work has shown that living in higher SES areas has a protective effect against increasing cardiometabolic risk [63] and that area deprivation is positively related with MetS and chronic inflammation [16]. Also, in an earlier analysis of baseline AusDiab data, area SES was negatively related to WC, TG and FBG, and positively related to HDL-C [64].

Area SES is deemed to impact MetS and its components by facilitating engagement in health-enhancing behaviours, including leisure-time physical activity and healthy eating [65–67], and by sometimes being associated with lower levels of air pollution [68, 69]. Higher SES neighbourhoods typically provide better access to healthy foods, recreational facilities and aesthetically-pleasing, safe environments that are conducive to recreational walking [70, 71]. Socio-economically advantaged neighbourhoods are also likely to host more educated, health-conscious residents that help others to adopt and sustain a healthy lifestyle [65]. As, apart from parks, this study did not measure access to recreational destinations, food outlets or examined neighbourhood attributes typically associated with SES and healthy lifestyles (aesthetics, safety and healthy foods) [65], the total (mediator-unadjusted) and direct, independent (fully-adjusted) effects of area SES on the membership to metabolic profiles were similar. It is interesting that measures of air pollution did not seem to explain the effects of area SES on metabolic profiles. In this regard, a study conducted in Sydney, Australia did not find a significant association between traffic-related air pollution and neighbourhood SES [72], while we found a positive association between average annual concentrations of NO_2_ and area SES in another study using data from the AusDiab 3 cohort [41]. Interestingly, area SES did not explain membership to metabolic profiles with vs. without MetS characterised by a high probability of high TG and large WC (LC3 No MetS vs. LC3 MetS). This is likely due to area SES generally showing a strong negative association with TG [64] and the LC3 No MetS profile being typified by a higher probability of high TG than both metabolic profiles with MetS (i.e., LC2 MetS and LC3 MetS). How neighbourhood SES affects MetS and its profiles and components remains an issue that future studies need to clarify in order to help address inequalities in cardiometabolic health.

### Ambient air pollution

As expected, higher average annual concentrations of PM_2.5_ were associated with higher odds of membership to the least healthy (LC3 MetS) vs. the healthiest metabolic profile (LC1 No MetS). Studies into the biological pathways of PM_2.5_ influences on metabolic health have shown that exposure to this air pollutant may generate oxygen-centred radicals that contribute to insulin resistance and vascular disease [73, 74], and activate cell-signalling pathways implicated in insulin resistance [75] and lipogenesis [76]. In a cohort of older men living in North-East USA, increases in levels of PM_2.5_ were associated with higher risk of developing MetS [23]. The associations were significant even when PM_2.5_ concentrations were below the USA Environmental Protection Agency’s health safety limit [23]. Also, borderline positive significant associations of PM_2.5_ with MetS incidence were found in 45–75 year-old individuals from German cities [22]. However, in contrast to previous research, our study examined the potential effects of PM_2.5_ adjusted for built environment and area SES confounders that are potential sources of air pollution, and also, albeit in another analysis of AusDiab3, attributes that promote behaviours beneficial to metabolic health [24]. That in this study long-term PM_2.5_ exposure could only differentiate between participants belonging to one of the six examined pairs of metabolic profiles with and without MetS (LC3 MetS vs. LC1 No MetS) suggests that, in the context of relatively low-pollution urban environments found in Australia, PM_2.5_ may be of particular relevance to abdominal adiposity (large WC) and dyslipidaemia (low HDL-C and high TG). In fact, in this study, PM_2.5_ was unable to distinguish between metabolic profiles with and without MetS characterised by a high probability of dyslipidaemia and large WC, or between the healthiest metabolic profile (LC1 No MetS) and the profile with MetS but without dyslipidaemia (LC2 MetS). In support of these findings, recent studies have reported positive associations of long-term PM_2.5_ with TG and WC [77, 78] and negative associations with HDL-C in middle-aged and older adults [78, 79].

The associations between average annual concentrations of NO_2_ and the odds of membership to metabolic profiles were weak and mixed. Whilst, as expected [22], NO_2_ exposure tended to be associated with higher odds of membership to the LC2 MetS profile than the LC2 No MetS profile, the opposite effect was found when comparing the LC2 MetS profile with the LC3 No Mets profile. The latter findings in our study might be due to the low levels of NO_2_ observed in the AusDiab3 cohort (median: 5.3 ppb compared to 9.1 ppb in a large European study [80]) and to environmental confounders not adequately controlling for the presence of environmental attributes that support an active lifestyle (e.g., access to destinations of daily living). In fact, NO_2_ can be seen as a proxy for the presence of human activity (e.g., retail and various food outlets) and accompanying traffic. As such, NO_2_ may be a predictor of active transport, a type of physical activity, which is negatively associated with MetS [81–83] and MetS components [84]. An increase in NO_2_ may indicate better access to facilities and, hence, higher levels of physical activity accumulated through active transport that may counteract the negative effect of NO_2_ on metabolic health, especially if NO_2_ levels are not too high and the level of access to destinations is sufficient to support health-enhancing levels of transport-related physical activity.

### Built environment

Only three built environmental attributes showed associations with metabolic profiles: percentage of commercial land, land use mix and street intersection density. As expected, having a higher percentage of commercial land in the neighbourhood was associated with a lower likelihood of membership to the LC2 MetS than the LC2 No MetS profile in both total and direct effect, fully-adjusted models. These associations were weaker when comparing LC2 MetS with the healthiest metabolic profile (LC1 No Mets). These weak and inconsistent associations may have been due to the variety of destinations classified within commercial land use having mixed effects on dietary behaviours associated with MetS components. For example, in a study of 5688, 50–74 year-old New Jersey residents, densities of fast-food establishments and storefronts were positively associated with obesity, whereas, density of supermarkets was not [85]. Consumption of fast-food meals has been associated with higher levels of obesity, FBG and BP [86, 87]. Clearly, future studies need to gain a better understanding of the contribution of various types of commercial destinations to health-enhancing behaviours and metabolic health in individuals with and without MetS. The same applies to other types of ‘non-natural’ land uses given that this study did not find significant direct associations of land use mix with metabolic profiles and a recent Australian longitudinal study failed to find a significant association between land use mix and WC [13].

We found only a weak positive association between a measure of non-commercial land use mix (including industrial land) and the odds of membership to the LC2 MetS profile vs. the healthiest metabolic profile (LC1 No MetS). Similar findings were observed for street intersection density when comparing the unhealthiest metabolic profile (LC3 MetS) with the healthiest profile (LC1 No MetS). However, these associations were no longer significant after accounting for other environmental attributes, including air pollution and commercial land. While this built environmental attribute is thought to benefit health by promoting active transport [7, 88, 89], it is also potentially associated with higher levels of traffic-related air pollution and greater exposure to air pollutants [90–92], which may explain why adjustment for NO_2_ and PM_2.5_ concentrations attenuated its positive association with metabolic profiles. Previous studies have reported conflicting findings about the potential effects of street intersection density on MetS components [63, 93–95]. None of these studies examined the potential contribution of air pollution in explaining these associations. Street intersection density and land use mix can be indicators of beneficial (access to services promoting healthful behaviours) as well as harmful (air pollution) influences on metabolic health. To better understand its impact on MetS and its components, future studies need to focus on disentangling the various antagonistic pathways through which it influences behaviours and health.

The above recommendations also hold for population density which, although unrelated to metabolic profiles in this study, is the main driver of changes in the built and natural environment [25, 96]. As such, it is likely to impact on metabolic health through other environmental characteristics. For example, population density has been found to lead to higher street intersection density and levels of PM_2.5_ [25, 97, 98], which in the current study were predictive of less healthy metabolic profiles. It has also been associated with lower levels of greenness [25] but better access to commercial services [25]. Population density may lead to some environmental changes that are beneficial to metabolic health (e.g., accessibility of services, availability of healthy foods, better health services) and other that are detrimental (e.g., lack of green space, air pollution). Thus, it is not surprising that previous studies have reported mixed findings in relation to its potential effects on MetS components [94, 96].

### Natural environment

Because access to greenspace, such as parkland, is thought to promote leisure-time physical activity [8] and be associated with lower levels of air pollution [98], negative associations between parkland and membership to less healthy vs. healthier metabolic profiles were expected in this study [99]. However, no such associations were observed. Previous studies have reported negative as well as nil associations between greenness and MetS [17, 100]. Similar findings were also observed for components of MetS. For example, greenness was negatively related with WC in recent European [17, 101] but not in Australian studies [13]. A recent systematic review and meta-analysis on this topic concluded that while access to greenspace is likely to be associated with lower odds of overweight/obesity, the evidence varies across measures of greenness and studies [102]. Only normalised difference vegetation index (NDVI) resulted in significant pooled associations, while percentage of greenspace, distance to greenspace and number of parks in the area did not. Mixed findings on the beneficial effect of greenness have been also reported in relation to BP [14, 103–105] and FBG [106] and HDL-C [14, 17, 107]. To better characterise the impact of greenness on MetS and its components, future studies would need to capture aspects of this environmental attribute that may be directly relevant to the hypothesised mechanisms of influence. These may include actual greenness (e.g., NDVI), since parks vary in their amount of greenness and this is an aspect that may impact on pollution; presence of trees and shade providing protection from the sun and heat; and quality and safety of green spaces.

Lastly, this study also examined the associations of percentage of blue space with MetS status and metabolic profiles because this neighbourhood attribute might facilitate engagement in physical activity [41] which, in turn, is beneficial to metabolic health [81–84]. However, we did not find significant effects. While no studies have specifically examined percentage of blue space as a correlate of MetS, Li and colleagues [106] found a negative association between distance to blue space and FBG in rural China. Clearly, the effect of access to blue space within the neighbourhood on metabolic health remains understudied and warrants further examination.

### Implications of findings

By examining total and direct associations of a wide range of urban neighbourhood environmental attributes with MetS status and metabolic profiles, this study has identified several findings with implications for future research as well as helping to inform public health and urban planning policy and practice. The first implication pertains to the measurement of environmental exposures. Urbanisation is a major global demographic phenomenon that is associated with poorer air quality and better access to services and opportunities for activities that may benefit or harm metabolic health. To understand how urban neighbourhood environments affect metabolic health and devise effective interventions and policies, there is a need to disentangle factors that are beneficial from those that are harmful. This requires a sufficiently precise characterisation of the built and natural environment that matches the mechanisms hypothesised to be responsible for the effects. This study suggests that fine-grained measures of destination accessibility associated with healthful or unhealthy behaviours (e.g., access to fast-food outlets, grocery stores, recreational facilities, good quality and safe green spaces) rather than coarse measures of land use are needed to accurately examine the impact of urban environments on MetS and their components.

The second study implication pertains to air pollution. Although, as evidenced in this study, air pollution levels in Australia are relatively low [108], our study suggests that they have detrimental effects on metabolic health that warrant environmental mitigation strategies, such as the promotion of active transport and public transport [109, 110], which, in turn, requires levels of densification that make these modes of transport feasible and more attractive than private motorised transport [111]. Sprawling neighbourhoods are highly prevalent in Australian cities [112] and appropriate urban planning policies are needed to stop this trend.

It is also noteworthy that area SES was a strong correlate of MetS status and metabolic profiles, which suggests that low income neighbourhoods should be targeted in public health interventions aimed at improving population-level metabolic health. However, the ways in which area SES contributes to social inequalities in metabolic health remain poorly understood and warrant further investigation.

### Strengths and limitations

The main strengths of this study include: the analyses of data from a national sample; recruited from 42 areas representative of Australian urban communities; the examination of curvilinearity of associations; adjustment for neighbourhood self-selection; the inclusion of a broad range of environmental variables capturing aspects of the built environment, natural environment and air pollution; and accounting for inter-relationships between environmental variables in the estimation of total and direct effects on MetS status and metabolic profiles. Among the main study limitations are the cross-sectional nature of the data, the utilisation of environmental measures that are insufficiently precise to accurately distinguish between healthful and harmful aspect of the urban environment, the lack of information on other activity spaces (outside the residential neighbourhood) or the time participants typically spent in their neighbourhood, and AusDiab3 being an opportunistic, potentially select sample. The imprecise measurement of environmental attributes, such as types of destinations, might have resulted in residual confounding and, hence, biased estimates of the direct, independent effects of the built environment and ambient air pollution on metabolic health. Additionally, two of the five metabolic profiles (LC3 No MetS and LC2 MetS) had a relatively small number of cases, hindering the identification of neighbourhood environmental correlates due to low statistical power.

## Conclusions

In this cohort study of Australian adults, area SES was the only neighbourhood environmental attribute associated with MetS status, with more advantaged neighbourhoods being predictive of better metabolic health. In contrast, in addition to area SES, three built environment attributes and ambient air pollution measures were associated with the odds of membership to specific metabolic profiles with MetS vs. without MetS. Environmental correlates of membership to profiles with vs. without MetS varied across pairs of profiles being compared, suggesting that the effects of environmental factors on various MetS components may differ. These findings support the utility of analyses of profiles of MetS components in conjunction with MetS status, or individual MetS components instead of MetS status in studies on environmental determinants of metabolic health. As expected, area SES and percentage of commercial land were negatively, and average annual concentrations of PM_2.5_ and NO_2_ were positively, associated with the odds of membership to less favourable metabolic profiles. The positive associations of land use mix and street intersection density with the odds of membership to less healthy metabolic profiles vanished after adjusting for environmental mediators (e.g., air pollution measures) demonstrating the need for comprehensive models of MetS examining all key inter-related environmental factors. Future research needs to consider conducting similar, ideally longitudinal, studies using environmental measures that more accurately characterise the neighbourhood environment in relation to behaviours or other mechanisms deemed to impact MetS and its components.

## Supplementary Information

**Additional file 1: Fig. A1.** Direct Acyclic Graph (DAG) depicting total effect of population density on MetS outcomes. **Table A1.** Model fit indices for latent class analyses (*N* = 3681). Material regarding participants with complete data. **Table A2.** Participant characteristics by latent class of MetS components. **Fig. A2.** Total effects of area SES on the odds of membership to metabolic profiles with MetS vs. without MetS.

## Data Availability

Data that support the findings of this study are available on request under a license agreement. Written applications can be made to the AusDiab Steering Committee (Dianna.Magliano@baker.edu.au).

## Abbreviations

AICM: Akaike Information Criterion Monte Carlo
AusDiab: Australian Diabetes, Obesity and Lifestyle Study
BICM: Bayesian Information Criterion Monte Carlo
BP: blood pressure
DAG: Directed Acyclic Graph
DIC: Deviance Information Criterion
FBG: fasting blood glucose
GAMs: generalized additive models
GIS: geographic information system
HDL-C: high-density lipoprotein cholesterol
IRSAD: Index of Relative Socioeconomic Advantage and Disadvantage
LC: latent class
LCA: latent class analysis
MetS: metabolic syndrome
NO_2_: nitrogen dioxide
PM_2.5_: particulate matter that has a diameter of 2.5 μm or smaller
SES: socio-economic status
TG: triglycerides
WC: waist circumference

## References

[1] Saklayen MG (2018). The global epidemic of the metabolic syndrome. Curr Hypertens Rep.

[2] O'Neill S, O'Driscoll L (2015). Metabolic syndrome: a closer look at the growing epidemic and its associated pathologies. Obes Rev.

[3] Alexander CM, Landsman PB, Teutsch SM (2003). NCEP-defined metabolic syndrome, diabetes, and prevalence of coronary heart disease among NHANES III participants age 50 years and older. Diabetes..

[4] Stevenson M, Thompson J, de Sá TH, Ewing R, Mohan D, McClure R (2016). Land use, transport, and population health: estimating the health benefits of compact cities. Lancet..

[5] GBD 2017 SDG Collaborators. Measuring progress from 1990 to 2017 and projecting attainment to 2030 of the health-related sustainable development goals for 195 countries and territories: a systematic analysis for the global burden of disease study 2017 [published correction appears in Lancet. 2019 Jun 22;393(10190):e44]. Lancet. 2018;392(10159):2091–2138. 10.1016/S0140-6736(18)32281-5. 10.1016/S0140-6736(18)32281-5 PMC6227911 30496107

[6] Barnett DW, Barnett A, Nathan A, Van Cauwenberg J, Cerin E; Council on environment and physical activity (CEPA) – older adults working group. Built environmental correlates of older adults' total physical activity and walking: a systematic review and meta-analysis. Int J Behav Nutr Phys Act 2017;14(1):103. 10.1186/s12966-017-0558-z. 10.1186/s12966-017-0558-z PMC5547528 28784183

[7] Cerin E, Nathan A, van Cauwenberg J, Barnett DW, Barnett A; Council on environment and physical activity (CEPA) – older adults working group. The neighbourhood physical environment and active travel in older adults: a systematic review and meta-analysis. Int J Behav Nutr Phys Act 2017;14(1):15. 10.1186/s12966-017-0471-5. 10.1186/s12966-017-0471-5 PMC5294838 28166790

[8] Van Cauwenberg J, Nathan A, Barnett A, Barnett DW, Cerin E (2018). Council on environment and physical activity (CEPA)-older adults working group. Relationships between neighbourhood physical environmental attributes and older adults' leisure-time physical activity: a systematic review and meta-analysis. Sports Med.

[9] McInerney M, Csizmadi I, Friedenreich CM, Uribe FA, Nettel-Aguirre A, McLaren L (2016). Associations between the neighbourhood food environment, neighbourhood socioeconomic status, and diet quality: an observational study. BMC Public Health.

[10] Gilham K, Gu Q, Dummer TJB, Spinelli JJ, Murphy RA (2020). Diet quality and neighborhood environment in the Atlantic Partnership for Tomorrow's health project. Nutrients..

[11] Assah FK, Ekelund U, Brage S, Mbanya JC, Wareham NJ (2011). Urbanization, physical activity, and metabolic health in sub-Saharan Africa. Diabetes Care.

[12] Salas-Salvadó J, Guasch-Ferré M, Lee CH, Estruch R, Clish CB, Ros E (2015). Protective effects of the Mediterranean diet on type 2 diabetes and metabolic syndrome. J Nutr.

[13] Carroll SJ, Dale MJ, Taylor AW, Daniel M (2020). Contributions of multiple built environment features to 10-year change in body mass index and waist circumference in a south Australian middle-aged cohort. Int J Environ Res Public Health.

[14] Cerin E, Barnett A, Shaw JE, Martino E, Knibbs LD, Tham R (2022). Urban neighbourhood environments, cardiometabolic health and cognitive function: a national cross-sectional study of middle-aged and older adults in Australia. Toxics..

[15] Yim E, Lee K, Park I, Lee S (2020). The prevalence of metabolic syndrome and health-related behavior changes: the Korea National Health Examination Survey. Healthcare..

[16] Keita AD, Judd SE, Howard VJ, Carson AP, Ard JD, Fernandez JR (2014). Associations of neighborhood area level deprivation with the metabolic syndrome and inflammation among middle- and older- age adults. BMC Public Health.

[17] de Keijzer C, Basagaña X, Tonne C, Valentín A, Alonso J, Antó JM (2019). Long-term exposure to greenspace and metabolic syndrome: a Whitehall II study. Environ Pollut.

[18] Baldock K, Paquet C, Howard N, Coffee N, Hugo G, Taylor A (2012). Associations between resident perceptions of the local residential environment and metabolic syndrome. J Environ Public Health.

[19] Yu Y, Paul K, Arah OA, Mayeda ER, Wu J, Lee E (2020). Air pollution, noise exposure, and metabolic syndrome - a cohort study in elderly Mexican-Americans in Sacramento area. Environ Int.

[20] Zang S-T, Luan J, Li L, Wu QJ, Chang Q, Dai HX (2021). Air pollution and metabolic syndrome risk: evidence from nine observational studies. Environ Res.

[21] Yang BY, Qian ZM, Li S, Fan S, Chen G, Syberg KM (2018). Long-term exposure to ambient air pollution (including PM1) and metabolic syndrome: the 33 communities Chinese health study (33CCHS). Environ Res.

[22] Matthiessen C, Lucht S, Hennig F, Ohlwein S, Jakobs H, Jöckel KH (2018). Long-term exposure to airborne particulate matter and NO2 and prevalent and incident metabolic syndrome - results from the Heinz Nixdorf recall study. Environ Int.

[23] Wallwork RS, Colicino E, Zhong J, Kloog I, Coull BA, Vokonas P (2017). Ambient fine particulate matter, outdoor temperature, and risk of metabolic syndrome. Am J Epidemiol.

[24] Cerin E (2019). Building the evidence for an ecological model of cognitive health. Health Place..

[25] Cerin E, Barnett A, Zhang CJP, Lai PC, Sit CHP, Lee RSY (2020). How urban densification shapes walking behaviours in older community dwellers: a cross-sectional analysis of potential pathways of influence. Int J Health Geogr.

[26] Dunstan DW, Zimmet PZ, Welborn TA, Cameron AJ, Shaw J, de Courten M, et al. The Australian diabetes, obesity and lifestyle study (AusDiab)--methods and response rates. Diabetes Res Clin Pract 2002;57:119–129. 10.1016/s0168-8227(02)00025-6 12062857

[27] Tanamas SK, Magliano DJ, Lynch BM, Sethi P, Willenberg L, Polkinghorne KR (2013). AusDiab 2012: the Australian diabetes, obesity and lifestyle study.

[28] Ho K, Jamsen KM, Bell JS, Korhonen MJ, Mc Namara KP, Magliano DJ (2018). Demographic, clinical and lifestyle factors associated with high-intensity statin therapy in Australia: the AusDiab study. Eur J Clin Pharmacol.

[29] White IR, Carlin JB (2010). Bias and efficiency of multiple imputation compared to complete-case analysis for missing covariate values. Stat Med.

[30] Alberti KGMM, Eckel RH, Grundy SM, Zimmet PZ, Cleeman JI, Donato KA (2009). Harmonizing the metabolic syndrome: a joint interim statement of the international diabetes federation task force on epidemiology and prevention; National Heart, Lung, and Blood Institute; American Heart Association; world heart federation; international atherosclerosis society; and International Association for the Study of obesity. Circulation..

[31] Adams MA, Frank LD, Schipperijn J, Smith G, Chapman J, Christiansen LB (2014). International variation in neighborhood walkability, transit, and recreation environments using geographic information systems: the IPEN adult study. Int J Health Geogr.

[32] Cerin E, Conway TL, Cain KL, Kerr J, De Bourdeaudhuij I, Owen N (2013). Sharing good NEWS across the world: developing comparable scores across 12 countries for the neighborhood environment walkability scale (NEWS). BMC Public Health.

[33] Australia Bureau of Statistics. IRSAD, Census of Population and Housing: Socio-Economic Indexes for Areas (SEIFA), Australia, (cat. no. 2033.0.55.001) Canberra: Australian Bureau of Statistics; 2011.

[34] Frank LD, Andresen MA, Schmid TL (2004). Obesity relationships with community design, physical activity, and time spent in cars. Am J Prev Med.

[35] Gaio V, Roquette R, Dias CM, Nunes B (2019). Ambient air pollution and lipid profile: systematic review and meta-analysis. Environ Pollut.

[36] Knibbs LD, Hewson MG, Bechle MJ, Marshall JD, Barnett AG (2014). A national satellite-based land-use regression model for air pollution exposure assessment in Australia. Environ Res.

[37] Knibbs LD, Coorey CP, Bechle MJ, Cowie CT, Dirgawati M, Heyworth JS (2016). Independent validation of national satellite-based land-use regression models for nitrogen dioxide using passive samplers. Environ Sci Technol.

[38] Knibbs LD, van Donkelaar A, Martin RV, Bechle MJ, Brauer M, Cohen DD (2018). Satellite-based land-use regression for continental-scale long-term ambient PM_2.5_ exposure assessment in Australia. Environ Sci Technol.

[39] Cerin E, Leslie E, du Toit L, Owen N, Frank LD (2007). Destinations that matter: associations with walking for transport. Health Place.

[40] Lamb KE, Thornton LE, King TL, Ball K, White SR, Bentley R (2020). Methods for accounting for neighbourhood self-selection in physical activity and dietary behaviour research: a systematic review. Int J Behav Nutr Phys Act.

[41] Cerin E, Barnett A, Shaw JE, Martino E, Knibbs LD, Tham R (2021). From urban neighbourhood environments to cognitive health: a cross-sectional analysis of the role of physical activity and sedentary behaviours. BMC Public Health.

[42] Collins LM, Lanza ST (2010). Latent class and latent transition analysis with application in the social, behavioral, and health sciences.

[43] White A, Murphy TB. BayesLCA: An R package for Bayesian latent class analysis. J Stat Softw, 2014;61(13), 1–28. http://www.jstatsoft.org/v61/i13/. Accessed 2 June 2021.

[44] Goodman LA (1974). Exploratory latent structure analysis using both identifiable and unidentifiable models. Biometrika..

[45] Galindo Garre F, Vermunt JK (2006). Avoiding boundary estimates in latent class analysis by Bayesian posterior mode estimation. Behaviometrika..

[46] Spiegelhalter DJ, Best NG, Carlin BP, van der Linde A (2002). Bayesian measures of model complexity and fit. J R Stat Soc Series B.

[47] Raftery AE, Newton MA, Satagopan JM, Krivitsky PN, Bernardo JM, Bayarri MJ, Berger JO, Dawid AP, Heckerman D, Smith AFM, West M (2007). Estimating the integrated likelihood via posterior simulation using the harmonic mean identity. Bayesian statistics.

[48] Nylund KL, Asparouhov T, Muthen BO (2007). Deciding on the number of classes in latent class analysis and growth mixture modelling: a Monte Carlo simulation study. Struct Equ Modeling.

[49] Adams MA, Sallis JF, Conway TL, Frank LD, Saelens BE, Kerr J (2012). Neighborhood environment profiles for physical activity among older adults. Am J Health Behav.

[50] Boakye-Dankwa E, Nathan A, Barnett A, Busija L, Lee RSY, Pachana N (2019). Walking behaviour and patterns of perceived access to neighbourhood destinations in older adults from a low-density (Brisbane, Australia) and an ultra-dense city (Hong Kong, China). Cities..

[51] R Core Team. R: a language and environment for statistical computing. Vienna, Austria: R Foundation for Statistical Computing. https://www.R-project.org/. Accessed 3 Apr 2021.

[52] Textor J, van der Zander B, Gilthorpe MK, Liskiewicz M, Ellison GTH (2016). Robust causal inference using directed acyclic graphs: the R package 'dagitty'. Int J Epidemiol.

[53] Wood S (2017). Generalized additive models: an introduction with R.

[54] Burnham KP, Anderson DR (2002). Model selection and multimodel inference: a practical information-theoretic approach.

[55] Michels KB, Rosner RA (1996). Data trawling: to fish or not to fish. Lancet..

[56] Rothman KJ (1990). No adjustments are needed for multiple comparisons. Epidemiology..

[57] Riahi SM, Moamer S, Namdari M, Mokhayeri Y, Pourhoseingholi MA, Hashemi-Nazari SS (2018). Patterns of clustering of the metabolic syndrome components and its association with coronary heart disease in the multi-ethnic study of atherosclerosis (MESA): a latent class analysis. Int J Cardiol.

[58] Boyko EJ, Doheny RA, McNeely MJ, Kahn SE, Leonetti DL, Fujimoto WY (2010). Latent class analysis of the metabolic syndrome. Diabetes Res Clin Pract.

[59] Liu X, Tao L, Cao K, Wang Z, Chen D, Guo J (2015). Association of high-density lipoprotein with development of metabolic syndrome components: a five-year follow-up in adults. BMC Public Health.

[60] Wang XR, Song GR, Li M, Sun HG, Fan YJ, Liu Y (2018). Longitudinal associations of high-density lipoprotein cholesterol or low-density lipoprotein cholesterol with metabolic syndrome in the Chinese population: a prospective cohort study. BMJ Open.

[61] Fong KC, Hart JE, James P (2018). A review of epidemiologic studies on greenness and health: updated literature through 2017. Curr Environ Health Reports.

[62] Mlambo P, Kengne AP, De Villiers A, Lambert EV, Puoane T (2016). Built environment, selected risk factors and major cardiovascular disease outcomes: a systematic review. PLoS One.

[63] Carroll SJ, Dale MJ, Niyonsenga T, Taylor AW, Daniel M (2020). Associations between area socioeconomic status, individual mental health, physical activity, diet and change in cardiometabolic risk amongst a cohort of Australian adults: a longitudinal path analysis. PLoS One.

[64] Williams ED, Magliano DJ, Zimmet PZ, Kavanagh AM, Stevenson CE, Oldenburg BF (2012). Area-level socioeconomic status and incidence of abnormal glucose metabolism: the Australian diabetes, obesity and lifestyle (AusDiab) study. Diabetes Care.

[65] Cerin E, Leslie E (2008). How socio-economic status contributes to participation in leisure-time physical activity. Soc Sci Med.

[66] Zhu Y, Duan MJ, Riphagen IJ, Minovic I, Mierau JO, Carrero JJ (2022). Separate and combined effects of individual and neighbourhood socio-economic disadvantage on health-related lifestyle risk factors: a multilevel analysis. Int J Epidemiol.

[67] Grant TL, Edwards N, Sveistrup H, Andrew C, Egan M (2010). Inequitable walking conditions among older people: examining the interrelationship of neighbourhood socio-economic status and urban form using a comparative case study. BMC Public Health.

[68] Crouse DL, Ross NA, Goldberg MS (2009). Double burden of deprivation and high concentrations of ambient air pollution at the neighbourhood scale in Montreal, Canada. Soc Sci Med.

[69] Næss Ø, Piro FN, Nafstad P, Smith GD, Leyland AH (2007). Air pollution, social deprivation, and mortality: a multilevel cohort study. Epidemiology..

[70] Cerin E, Frank LD, Sallis JF, Saelens BE, Conway TL, Chapman JE (2011). From neighborhood design and food options to residents' weight status. Appetite..

[71] McNeill LH, Kreuter MW, Subramanian SV (2006). Social environment and physical activity: a review of concepts and evidence. Soc Sci Med.

[72] Cowie CT, Ding D, Rolfe MI, Mayne DJ, Jalaludin B, Bauman A (2016). Neighbourhood walkability, road density and socio-economic status in Sydney, Australia. Environ Health.

[73] Houstis N, Rosen ED, Lander ES (2006). Reactive oxygen species have a causal role in multiple forms of insulin resistance. Nature..

[74] Perticone F, Ceravolo R, Candigliota M, Ventura G, Iacopino S, Sinopoli F (2001). Obesity and body fat distribution induce endothelial dysfunction by oxidative stress: protective effect of vitamin C. Diabetes..

[75] Sun Q, Yue P, Deiuliis JA, Lumeng CN, Kampfrath T, Mikolaj MB (2009). Ambient air pollution exaggerates adipose inflammation and insulin resistance in a mouse model of diet-induced obesity. Circulation..

[76] Mendez R, Zheng Z, Fan Z, Rajagopalan S, Sun Q, Zhang K (2013). Exposure to fine airborne particulate matter induces macrophage infiltration, unfolded protein response, and lipid deposition in white adipose tissue. Am J Transl Res.

[77] Zhang N, Wang L, Zhang M, Nazroo J (2019). Air quality and obesity at older ages in China: the role of duration, severity and pollutants. PLoS One.

[78] Yang BY, Bloom MS, Markevych I, Qian ZM, Vaughn MG, Cummings-Vaughn LA (2018). Exposure to ambient air pollution and blood lipids in adults: the 33 communities Chinese health study. Environ Int.

[79] Mao S, Chen G, Liu F, Li N, Wang C, Liu Y (2020). Long-term effects of ambient air pollutants to blood lipids and dyslipidemias in a Chinese rural population. Environ Pollut.

[80] Cai Y, Hansell AL, Blangiardo M, Burton PR, de Hoogh K, Doiron D (2017). Long-term exposure to road traffic noise, ambient air pollution, and cardiovascular risk factors in the HUNT and lifelines cohorts. Eur Heart J.

[81] Joseph RP, Vega-López S (2020). Associations of perceived neighborhood environment and physical activity with metabolic syndrome among Mexican-Americans adults: a cross sectional examination. BMC Res Notes.

[82] Werneck AO, Christofaro DGD, Ritti-Dias RM, Cucato GG, Conceição RDO, Santos RD (2020). Self-initiated changes in physical activity and incidence of metabolic syndrome: a longitudinal follow-up study. Diabetes Res Clin Pract.

[83] Zając-Gawlak I, Pelclová J, Groffik D, Přidalová M, Nawrat-Szołtysik A, Kroemeke A (2021). Does physical activity lower the risk for metabolic syndrome: a longitudinal study of physically active older women. BMC Geriatr.

[84] An KY (2020). Comparison between walking and moderate-to-vigorous physical activity: associations with metabolic syndrome components in Korean older adults. Epidemiol Health.

[85] Pruchno R, Wilson-Genderson M, Gupta AK (2014). Neighborhood food environment and obesity in community-dwelling older adults: individual and neighborhood effects. Am J Public Health.

[86] Chen L, Caballero B, Mitchell DC (2010). Reducing consumption of sugar-sweetened beverages is associated with reduced blood pressure: a prospective study among United States adults. Circulation..

[87] Pereira MA, Kartashov AI, Ebbeling CB, Van Horn L, Slattery ML, Jacobs DR (2005). Fast-food habits, weight gain, and insulin resistance (the CARDIA study): 15-year prospective analysis. Lancet..

[88] Bonaccorsi G, Manzi F, Del Riccio M, Setola N, Naldi E, Milani C (2020). Impact of the built environment and the neighborhood in promoting the physical activity and the healthy aging in older people: an umbrella review. Int J Environ Res Public Health.

[89] Sallis JF, Bowles HR, Bauman A, Ainsworth BE, Bull FC, Craig CL (2009). Neighborhood environments and physical activity among adults in 11 countries. Am J Prev Med.

[90] Zhang CJP, Barnett A, Johnston JM, Lai PC, Lee RSY, Sit CHP (2019). Objectively-measured neighbourhood attributes as correlates and moderators of quality of life in older adults with different living arrangements: the ALECS cross-sectional study. Int J Environ Res Public Health.

[91] Zhong J, Cai XM, Bloss WJ (2016). Coupling dynamics and chemistry in the air pollution modelling of street canyons: a review. Environ Pollut.

[92] Ishaque MM, Noland RB (2008). Simulated pedestrian travel and exposure to vehicle emissions. Transp..

[93] Araújo CAH, Giehl MWC, Danielewicz AL, Araujo PG, d'Orsi E, Boing AF (2018). Built environment, contextual income, and obesity in older adults: evidence from a population-based study. Cad Saude Publica.

[94] Hirsch JA, Moore KA, Barrientos-Gutierrez T, Brines SJ, Zagorski MA, Rodriguez DA (2014). Built environment change and change in BMI and waist circumference: multi-ethnic study of atherosclerosis. Obesity..

[95] Leonardi C, Simonsen NR, Yu Q, Park C, Scribner RA. Street connectivity and obesity risk: evidence from electronic health records. Am J Prev Med 2017;52(1S1):S40–7. 10.1016/j.amepre.2016.09.029. 10.1016/j.amepre.2016.09.029 27989291

[96] Liu M, Huang Y, Jin Z, Ma Z, Liu X, Zhang B (2017). The nexus between urbanization and PM_2.5_ related mortality in China. Environ Pollut.

[97] Carozzi F, Roth S. Dirty density: air quality and density of American cities. Bonn: IZA Institute of Labor Economics IZA DP No. 13191; 2020.

[98] James P, Hart JE, Laden F (2015). Neighborhood walkability and particulate air pollution in a nationwide cohort of women. Environ Res.

[99] Yang BY, Liu KK, Markevych I, Knibbs LD, Bloom MS, Dharmage SC (2020). Association between residential greenness and metabolic syndrome in Chinese adults. Environ Int.

[100] Voss S, Schneider A, Huth C, Wolf K, Markevych I, Schwettmann L (2021). Long-term exposure to air pollution, road traffic noise, residential greenness, and prevalent and incident metabolic syndrome: results from the population-based KORA F4/FF4 cohort in Augsburg, Germany. Environ Int.

[101] Sarkar C (2017). Residential greenness and adiposity: findings from the UK biobank. Environ Int.

[102] Luo YN, Huang WZ, Liu XX, Markevych I, Bloom MS, Zhao T (2020). Greenspace with overweight and obesity: a systematic review and meta-analysis of epidemiological studies up to 2020. Obes Rev.

[103] Bauwelinck M, Zijlema WL, Bartoll X, Vandenheede H, Cirach M, Lefebvre W (2020). Residential urban greenspace and hypertension: a comparative study in two European cities. Environ Res.

[104] Huang B, Xiao T, Grekousis G, Zhao H, He J, Dong G (2021). Greenness-air pollution-physical activity-hypertension association among middle-aged and older adults: evidence from urban and rural China. Environ Res.

[105] Poulsen MN, Schwartz BS, Nordberg C, DeWalle J, Pollak J, Imperatore G (2021). Association of greenness with blood pressure among individuals with type 2 diabetes across rural to urban community types in Pennsylvania, USA. Int J Environ Res Public Health.

[106] Li R, Chen G, Jiao A, Lu Y, Guo Y, Li S (2021). Residential green and blue spaces and type 2 diabetes mellitus: a population-based health study in China. Toxics..

[107] Twohig-Bennett C, Jones A (2018). The health benefits of the great outdoors: a systematic review and meta-analysis of greenspace exposure and health outcomes. Environ Res.

[108] Pandey KD, Wheeler D, Ostro B, Deichmann U, Hamilton K, Bolt K (2004). Ambient particulate matter concentrations in residential areas of world cities: new estimates based on global model of ambient particulates (GMAPS).

[109] Rodrigues PF, Alvim-Ferraz MCM, Martins FG, Saldiva P, Sá TH, Sousa SIV (2020). Health economic assessment of a shift to active transport. Environ Pollut.

[110] Xia T, Nitschke M, Zhang Y, Shah P, Crabb S, Hansen A (2015). Traffic-related air pollution and health co-benefits of alternative transport in Adelaide, South Australia. Environ Int.

[111] UN-Habitat. Planning and design for sustainable urban mobility: global report on human settlements 2013. London: Routledge; 2013.

[112] Coleman S. Built environment: increased urban footprint. In: Australia state of the environment. Australian Government Department of the Environment and Energy, Canberra. 2016. https://soe.environment.gov.au/theme/built-environment/topic/2016/increased-urban-footprint. 10.4226/94/58b65a5037ed8. Accessed 10 Sept 2021.

