# Supplementary material for: The neighbourhood environment and profiles of the metabolic syndrome
Source: Environ Health. 2022 Sep 3;21:80. doi: 10.1186/s12940-022-00894-4 (PMC9440568; doi:10.1186/s12940-022-00894-4)

**Additional file 1**

**Fig. A1** Direct Acyclic Graph (DAG) depicting total effect of population density on MetS outcomes

**
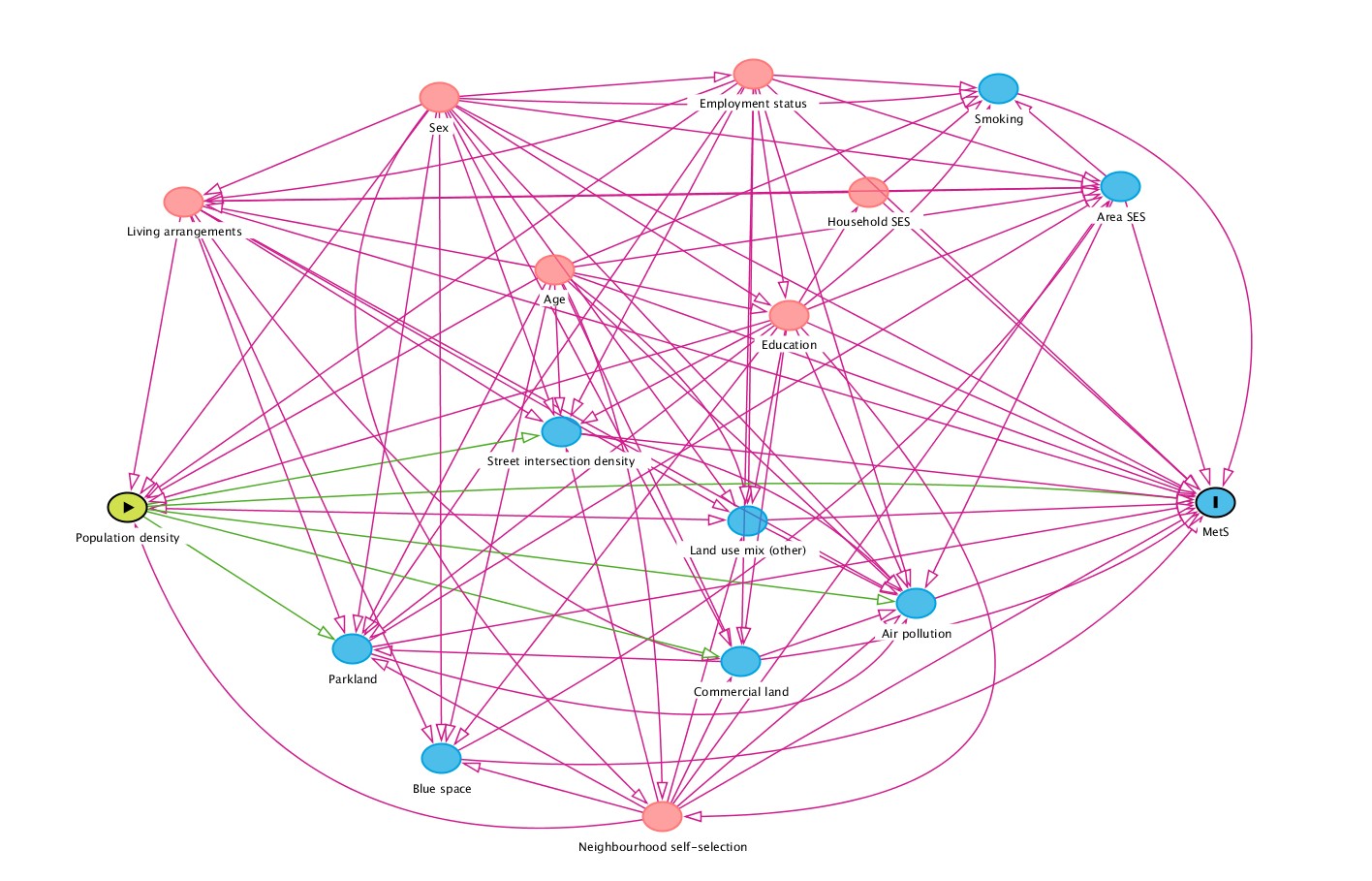
**

**Table A1** Model fit indices for latent class analyses (N = 3,681)

| # of latent classes | DIC | AICM | BICM |
| --- | --- | --- | --- |
| 1 | -20857.22 | -20857.37 | -20889.30 |
| 2 | -20072.75 | -20072.71 | -20139.78 |
| 3 | -19992.40 | -19999.27 | **-20114.04** |
| 4 | -19968.04 | -19991.67 | -20165.78 |
| 5 | **-19848.00** | **-19986.85** | -20158.57 |
| 6 | -19922.00 | -19988.70 | -20169.37 |

*Abbreviations:* *DIC* deviance information criterion, *AICM* Akaike’s information criterion Monte Carlo, *BICM* Bayesian information criterion Monte Carlo.

In bold, values indicating best fitting model.

**Material regarding participants with complete data**

Those with complete data were more likely to disclose their household income (*p*<.001), be younger (*p*<.001), live in higher SES areas (*p*=.010) and in neighbourhoods with higher NO_2_ (*p*=.003), PM_2.5_ (*p*=.028) and street intersection density (*p*≤.001), and lower population density (*p*≤.001).

**Table A2** Participant characteristics by latent class of MetS components

| **Characteristic** | **LC1** | **LC2** | **LC3** | ***p*** | **Sign. difference between pairs of LCs** |
| --- | --- | --- | --- | --- | --- |
| **Age**, years, M ± SD | 56.9 ± 10.6 | 63.9 ± 10.7 | 62.0 ± 11.0 | <.001 | LC1 vs. LC2; LC1 vs. LC3; LC2 vs. LC3 |
| **Sex**, female % | 63.8 | 48.9 | 51.4 | <.001 | LC1 vs. LC2; LC2 vs. LC3 |
| **Educational attainment, %** |  |  |  | <.001 |  |
| Up to secondary | 27.4 | 34.6 | 37.0 |  | LC1 vs. LC2; LC1 vs. LC3 |
| Trade, technician certificate | 27.9 | 29.6 | 30.3 |  |  |
| Associate diploma & equiv. | 14.3 | 15.2 | 14.7 |  |  |
| Bachelor degree, post-graduate diploma | 30.5 | 20.6 | 18.1 |  | LC1 vs. LC2; LC1 vs. LC3 |
| **Smoking history, %** |  |  |  | <.001 |  |
| Current smoker | 6.9 | 5.5 | 10.0 |  | LC1 vs. LC3; LC2 vs. LC3 |
| Previous smoker | 32.7 | 39.1 | 40.0 |  | LC1 vs. LC2; LC1 vs. LC3 |
| Non-smoker | 60.4 | 55.4 | 50.0 |  | LC1 vs. LC2; LC1 vs. LC3 |
| **Living arrangements, %** |  |  |  | <.001 |  |
| Couple without children | 44.0 | 55.9 | 48.0 |  | LC1 vs. LC2; LC2 vs. LC3 |
| Couple with children | 36.3 | 21.1 | 24.7 |  | LC1 vs. LC2; LC1 vs. LC3 |
| Other | 19.7 | 23.0 | 27.3 |  | LC1 vs. LC3 |
| **Household income, %** |  |  |  | <.001 |  |
| Up to $49,999 | 23.9 | 39.6 | 39.7 |  | LC1 vs. LC2; LC1 vs. LC3 |
| $50,000 - $99,999 | 30.5 | 26.9 | 25.7 |  | LC1 vs. LC3 |
| $100,000 and over | 38.5 | 25.2 | 25.2 |  | LC1 vs. LC2; LC1 vs. LC3 |
| Does not know or refusal | 7.2 | 8.3 | 9.4 |  |  |
| **Employment status, %** |  |  |  | <.001 |  |
| In paid work | 62.0 | 49.4 | 49.1 |  | LC1 vs. LC2; LC1 vs. LC3 |
| Volunteering | 16.0 | 15.9 | 17.2 |  |  |
| Neither | 22.0 | 34.7 | 33.8 |  | LC1 vs. LC2; LC1 vs. LC3 |
| **Neigh. self-selection – access to destinations**, M ± SD | 2.9 ± 1.2 | 2.9 ± 1.3 | 3.0 ± 1.3 | .739 |  |
| **Neigh. self-selection – recreational facilities**, M ± SD | 3.1 ± 1.4 | 3.0 ± 1.5 | 3.0 ± 1.5 | .264 |  |
| **Population density**, persons/ha, M ± SD | 17.5 ± 9.9 | 17.2 ± 10.6 | 17.4 ± 9.9 | .864 |  |
| **Commercial land use**, %, M ± SD | 2.4 ± 5.6 | 2.6 ± 7.2 | 2.8 ± 5.7 | .255 |  |
| **Parkland**, %, M ± SD | 12.2 ± 12.8 | 11.3 ± 11.9 | 11.6 ± 13.0 | .219 |  |
| **Blue space**, %, M ± SD | 0.3 ± 2.4 | 0.2 ± 1.8 | 0.2 ± 1.9 | .667 |  |
| **Street intersection density**, intersections/km^2^, M ± SD | 60.8 ± 32.3 | 63.4 ± 33.1 | 63.7 ± 32.7 | .051 |  |
| **Land use mix (other)**, M ± SD | 0.1 ± 0.1 | 0.1 ± 0.1 | 0.1 ± 0.1 | .118 |  |
| **Area SES (IRSAD)**, M ± SD | 6.8 ± 2.5 | 6.4 ± 2.7 | 6.0 ± 2.7 | <.001 | LC1 vs. LC2; LC1 vs. LC3; LC2 vs. LC3 |
| **Air pollution: NO_2_**, ppb, M ± SD | 5.7 ± 2.2 | 5.4 ± 2.0 | 5.6 ± 2.0 | .013 | LC1 vs. LC2 |
| **Air pollution: PM_2.5_, μg/m^3^,** M ± SD | 6.2 ± 1.7 | 6.3 ± 1.7 | 6.5 ± 1.7 | .006 | LC1 vs. LC3 |

*Abbreviations: M* mean, *SD* standard deviation, *IRSAD* Index of Relative Socioeconomic Advantage and Disadvantage, *NO_2_* nitrogen dioxide, *PM_2.5_* particulate matter < 2.5 µm, *ppb* parts per billion, *LC* latent class, *Sign.* statistically significant (*p*<.05), *p* probability value, *LC1* latent class “Lower probability of MetS components”, *LC2* latent class “Medium-to-high probability of high fasting blood glucose, waist circumference and blood pressure”, *LC3* latent class “Higher probability of MetS components”.

**Fig. A2** Total effects of area SES on the odds of membership to metabolic profiles with MetS vs. without MetS. Legend: Panel A represents the odds of membership to the LC2 MetS profile (“Medium-to-high probability of high fasting blood glucose, waist circumference and blood pressure & having MetS”) compared to the LC1 No MetS profile (“Lower probability of MetS components & not having MetS”). Panel B represents the odds of membership to the LC2 MetS profile compared to the LC2 No MetS profile (“Medium-to-high probability of high fasting blood glucose, waist circumference and blood pressure & not having MetS”)


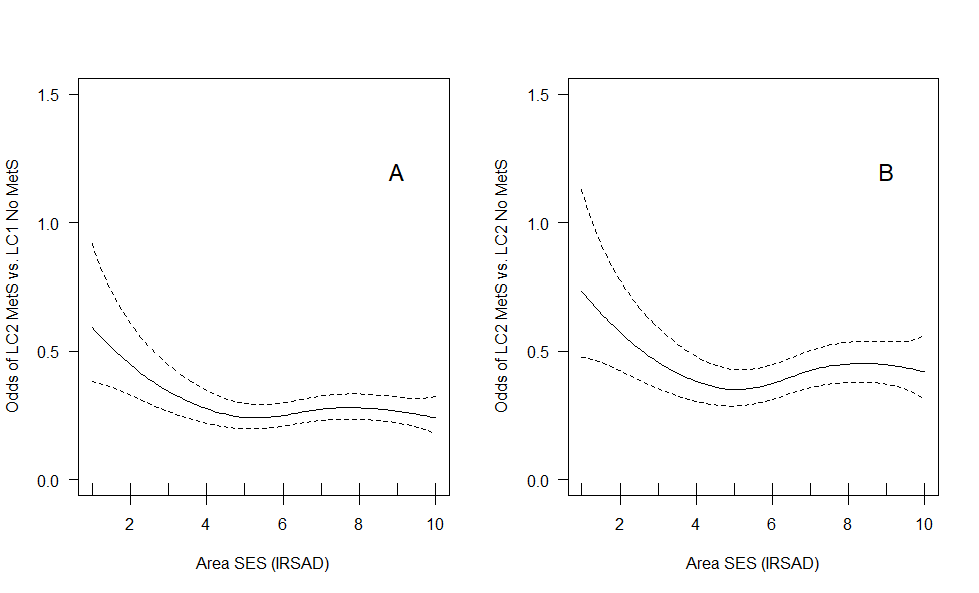

Supplement: Supplementary file 1 — Additional file 1: Fig. A1. Direct Acyclic Graph (DAG) depicting total effect of population density on MetS outcomes. Table A1. Model fit indices for latent class analyses (N = 3681). Material regarding participants with complete data. Table A2. Participant characteristics by latent class of MetS components. Fig. A2. Total effects of area SES on the odds of membership to metabolic profiles with MetS vs. without MetS. [file 12940_2022_894_MOESM1_ESM.docx]
